# microRNA-210 and microRNA-3570 Negatively Regulate NF-κB-Mediated Inflammatory Responses by Targeting RIPK2 in Teleost Fish

**DOI:** 10.3389/fimmu.2021.617753

**Published:** 2021-03-31

**Authors:** Hui Su, Renjie Chang, Weiwei Zheng, Yuena Sun, Tianjun Xu

**Affiliations:** ^1^ Laboratory of Fish Molecular Immunology, College of Fisheries and Life Science, Shanghai Ocean University, Shanghai, China; ^2^ Laboratory of Marine Biology and Biotechnology, Qingdao National Laboratory for Marine Science and Technology, Qingdao, China; ^3^ Key Laboratory of Exploration and Utilization of Aquatic Genetic Resources (Shanghai Ocean University), Ministry of Education, Shanghai, China; ^4^ National Pathogen Collection Center for Aquatic Animals, Shanghai Ocean University, Shanghai, China

**Keywords:** microRNAs, RIPK2, NF-κB, immune response, negative regulation, teleost fish

## Abstract

Pathogen infection can cause the production of inflammatory cytokines, which are key mediators that cause the host’s innate immune response. Therefore, proper regulation of immune genes associated with inflammation is essential for immune response. Among them, microRNAs (miRNAs) as gene regulator have been widely reported to be involved in the innate immune response of mammals. However, the regulatory network in which miRNAs are involved in the development of inflammation is largely unknown in lower vertebrates. Here, we identified two miRNAs from miiuy croaker (*Miichthys miiuy*), miR-210 and miR-3570, which play a negative regulatory role in host antibacterial immunity. We found that the expressions of miR-210 and miR-3570 were significantly upregulated under the stimulation of Gram-negative bacterium *vibrio harveyi* and LPS (lipopolysaccharide). Induced miR-210 and miR-3570 inhibit inflammatory cytokine production by targeting RIPK2, thereby avoiding excessive inflammation. In particular, we found that miR-210 and miR-3570 negatively regulate antimicrobial immunity by regulating the RIPK2-mediated NF-κB signaling pathway. The collective results indicated that both miRNAs are used as negative feedback regulators to regulate RIPK2-mediated NF-κB signaling pathway and thus play a regulatory role in bacteria-induced inflammatory response.

## Introduction

The innate immune system is the host’s initial defense system against the pathogens invasion. When pathogen infects the host, pattern recognition receptors (PRRs) in the cell membrane or cytoplasm can recognize and bind to pathogen-associate molecular patterns (PAMPs) on the surface of the pathogen, thereby triggering innate immune response (1, 2). At present, it has been widely reported that there are mainly two types of receptors involved in the detection of bacterial-specific components, Toll like receptors (TLRs) and nucleoside binding oligomerization domain (NOD)-like receptors (NLRs) (3, 4). Among, NLRs as one of the important cytoplasmic PRRs, which plays a vital role in the response to various pathogen infections, such as LPS, bacterial peptidoglycan fragment muramyl dipeptide (MDP) and G-D-glutamyl-meso-diaminopimelic acid (iE-DAP) (5–7). As the earliest to be studied NLR-related proteins, NOD1 and NOD2 can trigger a series of signal cascades by recruiting key adaptor protein RIPK2, thereby activating NF-κB transcription factors and promoting the production of inflammatory factors (8). However, the excessive activation the RIPK2-mediated antibacterial response can destroy the occurrence of immune homeostasis (9). Therefore, in order to ensure a moderate immune response, this signaling pathway should be precisely regulated.

Receptor interacting serine/threonine kinase 2 (RIPK2) is the downstream adaptor protein of NOD1 and NOD2 (8). RIPK2 was initially thought to be associated with TLR signal transduction (10). However, in RIPK2-deficient mice, Park et al. confirmed that RIPK2 can affect cell signaling transduction and cytokine production caused by NOD1 and NOD2, but not TLR (11). In teleost fish, RIPK2 is reported to be present in a variety of fish and has been shown to participate in the antibacterial immune response after pathogen infection (12, 13). Our previous reports have found that NOD1 plays an important role in recognizing lipopolysaccharide (LPS) and iE-DAP in miiuy croaker (7, 14). Due to RIPK2 is an adaptor protein downstream of NOD1. Thus, in-depth study of RIPK2-mediated signaling pathways may help clarify the resistance of fish to bacterial infections. In recent years, in teleost fish, a large number of microRNAs (miRNAs) have been identified to be endogenous negative regulators involved participate in the regulation of host immune signals (15). However, studies on miRNAs regulating RIPK2-mediated signaling pathways have not been reported yet in teleost. Consider that the core protein TLR4 which recognizes LPS is absent in most fish (16), while in teleost fish, NLRs as one of the few pattern recognition receptors that can recognize LPS (7), RIPK2 is a key gene in the NLRs signaling pathway. Therefore, it is urgent for us to explore whether there are miRNAs that can regulate RIPK2.

miRNAs as a class of highly conserved single-stranded RNA molecules with a length of about 20 to 24 nt, which can bind to the 3′UTR region of the mRNAs, and then inhibit mRNA translation or promote the mRNA degradation to regulate the gene expression (17, 18). miRNAs are highly conserved in different species and up to 60% of protein-coding genes are regulated by miRNAs (19). Therefore, miRNAs can be widely involved in the regulation of various biological processes, such as apoptosis, proliferation, differentiation, and immunity response (20–22). Increasing evidence shows that miRNAs can participate in the negative regulation of immune responses by directly targeting the expression of PRRs or PRRs-related adaptor protein (23, 24). For example, miR-21 inhibit production of Toll receptor-dependent antiviral genes by targeting the adaptor MyD88 and IRAK1 (23). MiR-150 eliminated NOD2-induced immune modulator production by targeting RIPK2 (24) Similarly, in teleost fish, miRNAs are regarded as a fine-tuning regulator of immune responses that could involve in bacterial or viral infection by targeting immune-related genes (15). For example, miR-217 targets TAK1 and NOD1, respectively, to regulate the immune response induced by bacterial infection (25, 26). In RLRs-mediated antiviral response, miR-122 negatively regulates MAVS to facilitate the immune escape of the virus (27). Although miRNAs regulating various TLRs and RLRs downstream adaptor proteins have been widely reported, the relationship between miRNAs and the downstream adaptor RIPK2 of NLRs has not been reported in teleost fish.

Teleost fish, as the representative population of vertebrates, is an important link in the evolution of invertebrates to vertebrates. Similar to mammals, it has a complete innate immune system containing conserved immune-related genes and a series of signaling events to respond to invasion by various pathogens (28). Therefore, they are considered to be excellent biological model for studying vertebrate immunology. Miiuy croaker (*Miichthys Miiuy*) is an important marine economic fish. Up to now, the transcriptome (29), whole genome (30), functional genome, and immune pathway regulation levels of this species have been reported (31), which can be used as a useful model for the immune response of fish. With the growing destruction of aquaculture waters, the cultivation of miiuy croaker is facing severe challenges from kinds of diseases. *Vibrio harveyi*, a Galanz-negative bacterium with strong infectivity, can cause high mortality and severe economic losses (32).

Previous studies have reported the expression of hundreds of miRNAs in the spleen of miiuy croaker treated with *V. harveyi* (26). However, miRNAs involved in the regulation of RIPK2 and its signaling pathway have not been found in fish during bacterial infection. In this study, we used miRNA target prediction program, we predicted two miRNAs, miR-210 and miR-3570, which target miiuy croaker RIPK2 gene, are significantly up-regulated after treatment with *V. harveyi*. We also found that overexpression of miR-210 and miR-3570 inhibited the production of inflammatory cytokines by targeting RIPK2. More importantly, the two miRNAs can regulate the antimicrobial response by inhibiting the NF-κB signaling pathway, thus avoiding the occurrence of excessive inflammation. To our knowledge, this is the first study to demonstrate that miRNAs could act as negative regulators to involved in RIPK2-mediated NF-κB signal pathway in teleost fish.

## Materials and Methods

### Animals and Challenge

Healthy miiuy croaker juveniles were obtained from Zhoushan Fisheries Research Institute (Zhejiang Province, China). It was cultured in aerated seawater tanks at 25°C for 3 months before the experiment. The bacterial challenge was carried out as described above (33), to put it simply, these healthy fish (about 50 g) were randomly divided into two groups of three fish in each group, for the stimulation experiment, miiuy croakers were intraperitoneal with 0.2 ml *V. harveyi* (1.5 × 10^8^ CFU/ml) or 0.2 mg suspension of LPS (about 5 mg/kg, Invitrogen), respectively. For comparison, 0.2 ml physiological saline was used to challenge the individuals. Then, fish are sacrificed at different time points; briefly, the fish were completely immersed in 100 mg/L anesthetic solution (MS-222, Sigma) for 10 min. After the gills stopped breathing and the belly rolled up, tissue samples are collected from the fish. The spleen tissues were collected from three individual at each time. All animal experiments procedures were conducted in accordance with the recommendations of National Institutes of Health’s Guide for the Care and Use of Laboratory Animals, and the experimental protocols were approved by the Research Ethics Committee of Shanghai Ocean University (no. SHOU-DW-2018-047).

### Cell Culture and LPS Exposure

To separate and obtain the MIC cells, intestine tissues from healthy miiuy croakers were collected and chopped, next, conduct sterile filtration by using cell filter with 100 µm pore size in L-15 medium ((HyClone, USA), which was contained 2% FBS (Gibco, USA), 100 U/ml penicillin (Gibco, USA), 100 µg/ml streptomycin (Gibco, USA), and 20 U/ml heparin (Solarbio, China). Then, the cell suspension was added into 51% Percoll (Pharmacia, USA) separating medium and centrifuged at the condition of 400 g at 4°C for 40 min. Next, the supernatant was removed and the cells were collected at interface, washed the cells twice with L-15 medium, and seeded in a 6-well plate at a density of about 4 × 10^7^ cells/well, the cells were then cultured in the incubator at 26°C with 4% CO_2_. Epithelioma papulosum cyprini cells (EPC) were placed in medium 199 (Invitrogen) supplemented with 10% FBS, 100 U/ml penicillin, and 100 mg/ml streptomycin at 26°C in 5% CO2.

For the LPS exposure, MIC cells and EPC cells were challenged with ultrapure LPS (2 μg/ml) and incubated for the different time as indicated. Untreated cells served as a control group, and each experiment had three biological replicates.

### Plasmid Construction

To construct miiuy croaker RIPK2-3′UTR reporter plasmid, the RIPK2-3′UTR region was amplified using PCR from cDNA of miiuy croaker and inserted into the pmir-GLO luciferase reporter vector (Promega) by using the *Sac* I and *Xba* I restriction sites. Similarly, the 3′UTR of *Larimichthys crocea* RIPK2 gene (*Lcr*RIPK2 3′UTR) was amplified and cloned into the *Nhe* I and *Xho* I restriction sites of the pmir-GLO vector. The mutant-type of RIPK2-3′UTR (RIPK2-3′UTR mut) reporter plasmid was obtained by using Mut Express II Fast Mutagenesis Kit V2 (Vazyme) with specific primers (**Supplemental Table 2**). Moreover, the miiuy croaker wild type of RIPK2-3′UTR or mutant-type was cloned into the mVenus-C1 vector (Invitrogen), which contains the sequence of enhanced green fluorescent protein (GFP). To construct the pre-miRNA vector, the pre-miR-3570 (33) and pre-miR-210 (34) sequence were amplified by PCR and cloned into pcDNA3.1 vector (Invitrogen). In order to construct the RIPK2 expression plasmid, the full length of coding sequence (CDS) and 3′UTR of miiuy croaker RIPK2 gene were amplified by specific primer pairs with Hind III and EcoR I endonuclease sites and inserted into pcDNA3.1 vector (Invitrogen) with the Flag tag. All the recombinant plasmids were extracted through Endotoxin-Free Plasmid DNA Miniprep Kit (Tiangen) and confirmed by Sanger sequencing.

### miR-210 and miR-3570 Target Gene Identification

The miR-210 and miR-3570 targets were predicted using Targetscan (35), miRanda (36), and MicroInspector (37) algorithms. Predictions were ranked by the predicted efficacy of targeting as calculated using the context and scores of the sites.

### miRNA Mimics and Inhibitors

miR-3570 mimics, miR-3570 inhibitors and miR-210 mimics, miR-210 inhibitors and control oligonucleotides relative sequence were examined in previous reports (33, 34).

### RNA Interference

The sequence of RIPK2 specific siRNA1 (si-RIPK2-1) were 5′-CACAAAGCCAGAUUCAACUACAUCA-3′ (sense) and 5′-UGAUGUAGUUGAAUCUGGUUUGUG-3′ (antisense). The RIPK2-specific siRNA2 (si-RIPK2-2) were 5′-CCAUCAAGUGCCUGAAAACUTT-3′(sense) and 5′-AGUUUCAGGCACUUGAUGGTT-3′(antisense). The sequence of RIPK2 specific siRNA3 (si-RIPK2-3) were 5′-GGGCUGAUGUGAAACACGAUAUGUA-3′ (sense) and 5′-UACAUAUCGUGUUUCACAUCAGCCC-3′ (antisense). The negative control siRNA sequences were (NC) 5′-UUCUCCGAACGUGUCACGUTT-3′ (sense) and 5′-ACGUGACACGUUCGGAGAATT-3′ (antisense).

### Cell Transfection and Dual-Luciferase Reporter Assays

Before transient transfection, the EPC cells or MIC cells were seeded into 24-well plates or 48-well plates and incubated overnight. Lipofectamine™ RNAiMAX (Invitrogen) was used for transient transfection NC, miRNA mimics, miRNA inhibitors or siRNA into MIC cells. DNA plasmid transfection was performed on the cells using Lipofectamine™ 3000 (Invitrogen) according to the manufacturer’s instructions.

For miRNA target identification, the wild-type or mutant-type of RIPK2-3′UTR luciferase reporter plasmid (100 ng) was contransfected with miR-3570 mimics 100 nM), miR-210 mimics (100 nM), and NC (100 nM) or the pre-miR-3570 (200 ng) and pre-miR-210 plasmid (200 ng) or pcDNA3.1 vector (200 ng) into EPC cells. To determine the functional regulation of miR-210 and miR-3570 during LPS stimulation, EPC cells were cotransfected with RIPK2 expression plasmid (500 ng), phRL-TK Renilla luciferase plasmid and NF-κB, IL-1β, and IL-8 luciferase report genes together with miR-3570 mimics, miR-210 mimics or controls mimics (NC) for dual-luciferase reporter assays. After cells were treated with LPS (2 μg/ml) for 6 h, the cells were lysed for reporter activity testing using the dual-luciferase reporter assay system (Promega) following the manufacturer’s instructions. All the luciferase activity values were achieved against the renilla luciferase control. Transfection of each conducted was performed in triplicate in each assay. Ratios of Renilla luciferase readings to firefly luciferase readings were taken for each experiment, and triplicates were averaged.

### RNA Extract and RT-qPCR

Total RNA was extracted with Trizol reagent (Invitrogen) according to the manufacturer’s protocol, and the cDNA was synthesized using the FastQuant RT Kit (Tiangen) which includes DNase treatment of RNA to eliminate genomic contamination. The small RNA was isolated with miRcute miRNA Isolation Kit (Tiangen Biotech), and miRcute miRNA FirstStrand cDNA Synthesis Kit (Tiangen) was applied to reverse transcription of miRNAs. All gene transcripts were measured by SYBR Premix Ex Taq™ (Takara) using a 7,500 real-time PCR system (Applied Biosystems). β-actin and 5.8S rRNA were employed as endogenous controls for mRNA and miRNA, respectively, as described. Primer sequences are displayed in **Supplementary Table 1**.

### Western Blotting Analysis

Cellular lysates were generated by using 1× SDS-PAGE loading buffer. The proteins were extracted from the cells, and the concentrations were measured with a BCA Protein Assay kit (Vazyme), then subjected to SDS-PAGE (10%) gel and transferred to PVDF (Millipore) membranes by semidry blotting (Bio-Rad Trans Blot Turbo System). The membranes were blocked with 5% BSA. The Proteins were blotted with different antibodies. Antibodies against RIPK2 was diluted at 1:400 (Abcam); anti-Flag and anti-β-actin monoclonal antibodies were diluted at 1:2,000 (Sigma, USA); HRP-conjugated anti-rabbit IgG or anti-mouse IgG (Abbkine) was diluted 1:5,000. The results are representative of three independent experiments. Immunoreactive proteins were detected using WesternBright™ ECL (Advansta). Digital imaging was performed by using cold charge-coupled device (CCD) camera. The Image J analysis was used to analyze the grayscale of the protein band and get the results of the gray value. The ratio of the gray value of target protein against the gray value of β-actin was the relative expression of protein.

### Statistical Analysis

All experiments were performed at least three independent times, with three replicates for each experiment. The relative gene expression data was acquired using the 2 ^-ΔΔCT^ method, Data on quantified relative expression of miR-210, miR-3570, RIPK2 and inflammatory factors genes in stimulated MIC cells, and unstimulated cells (0 h) were compared using an independent samples t-test for equality of means. Dual luciferase reporter gene analysis data and TNF-α, IL-1β, and IL-8 genes in stimulated and control cells at each time point was examined with one-way analysis of variance (ANOVA) followed by Duncan’s multiple comparison tests for their comparison (38). Results are expressed as means ± SE (standard error), and differences between means considered statistically significant at P values of <0.05.

## Results

### *V. harveyi* and LPS Increase the Expression of Two miRNAs

**Figure 1:**
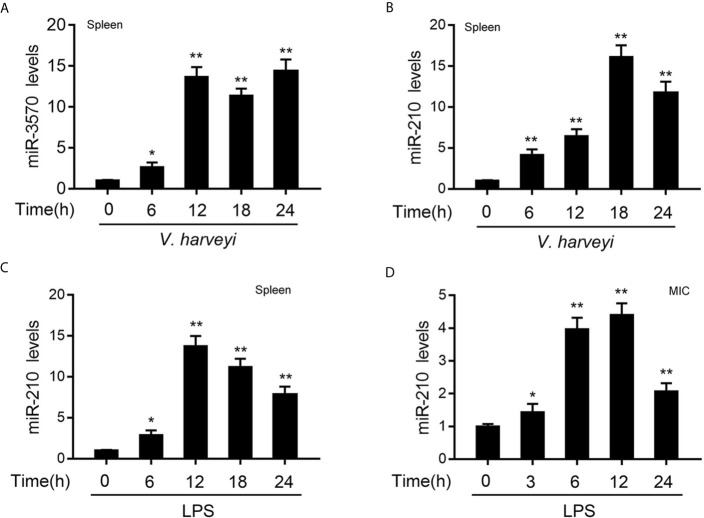
The expression profiles of miR-210 and miR-3570 following *V. harveyi* infection. **(A, B)** qPCR analysis of miR-3570 **(A)** and miR-210 **(B)** expression profiles in miiuy croaker spleen tissues treated with *V. harveyi* at the indicated time points. **(C, D)** qPCR for the abundance of miR-210 mRNA in miiuy croaker spleen tissues **(C)** and MIC cells **(D)** after LPS stimulation at the indicated time. The miR-210 and miR-3570 expression levels were all measured by qRT-PCR and normalized to 5.8S rRNA. Results are standardized to 1 in control samples. All data are presented as the means ± SE from three independent triplicate experiments. **p < 0.01; *p < 0.05 versus the controls.

In order to investigate the miRNAs that may be involved in the regulation of RIPK2 and its signaling pathway after bacterial infection. We used miiuy croaker RIPK2-3′UTR (untranslated region) as a query sequence to predict candidate miRNAs binding to RIPK2 through a miRNA target prediction program. Among the 34 predicted miRNAs, the differential expression of miR-210 and miR-3570 was particularly significant when infected with *V. harveyi*. To confirm the reliability of the predicted results, we examined the expression profiles of miR-210 and miR-3570 in miiuy croaker spleen tissues infected with *V. harveyi* by qRT-PCR. As shown in **Figures 1A, B**, the mRNA levels of miR-3570 and miR-210 were sharply increased in spleen, reaching peaks at 12 h and 18h upon infection, respectively. Since previous studies have reported that miR-3570 expression profiles can be upregulated in the spleen tissue and macrophages treatment with LPS (33). Here, we mainly focused on evaluating the expression profiles of miR-210 spleen tissues and MIC cells following LPS stimulation. The results showed that miR-210 was significantly up-regulated in the spleen tissues and reaches a peak at 12 h after treatment with LPS (**Figure 1C**). The expression profiles of miR-210 was also showed the same effect in MIC cells after LPS stimulation (**Figure 1D**). In summary, the expression of miR-210 and miR-3570 can be up-regulated by *V. harveyi* and LPS stimulation, indicating that both miRNAs involved in the regulation of immune response caused by Gram-negative bacteria infection.

### miR-210 and miR-3570 Reduced Inflammatory Cytokines Production

**Figure 2:**
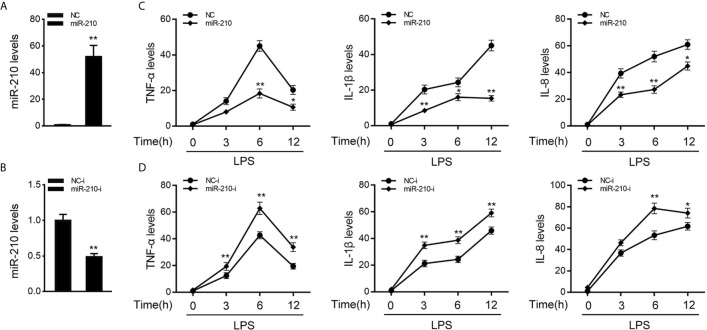
miR-210 negatively regulate expression of inflammatory cytokines. **(A, B)** The miiuy croaker MIC cells were transfected with control mimics (NC) and miR-210 mimics (miR-210) **(A)** or miR-210 inhibitors (miR-210-i) **(B)** and control inhibitors (NC-i) within a final concentration of 100 nM. After 48 h posttransfection, miR-210 expression was measured by qRT-PCR and normalized to 5.8S rRNA. **(C, D)** miR-210 or NC **(C)** and miR-210-i or NC-i **(D)** transfected into MIC cells for 48 h. MIC cells were then stimulated with LPS at different time points, then the mRNA expression levels of TNF-α, IL-1β, and IL-8 were analyzed by qPCR and normalized to β-actin. Results are standardized to 1 in control cells. All data are presented as the means ± SE from three independent triplicate experiments. **p < 0.01; *p < 0.05 versus the controls.

To investigate the function of miR-210 and miR-3570 in host antibacterial, we evaluated the effects of overexpression or inhibition of miR-210 and miR-3570 on the inflammatory cytokines in miiuy croaker MIC cells after LPS stimulate. In previous studies, we have confirmed that miR-3570 has a negative regulatory effect on inflammatory cytokines in LPS-treated macrophages (33), and we speculated that miR-3570 may show a similar effect in MIC cells. Therefore, we evaluated the effect of synthetic exogenous miR-210 and miR-3570 mimics and inhibitors on the expression of endogenous miR-210 and miR-3570 in MIC cells. miRNA mimics are chemically synthesized double-stranded RNAs (dsRNAs) that can simulate mature miRNAs, whereas miRNA inhibitors are chemically modified antisense single-stranded RNAs (ssRNAs) that block the endogenous miRNAs activity through base complementary. Thus, as expected, compared to control mimics (NC), the miR-210 and miR-3570 mimics markedly increased the expression of miR-210 and miR-3570 in MIC cells, respectively (**Figure 2A** and **Supplementary Figure 1A**). whereas the miR-210 and miR-3570 inhibitor reduced miR-210 and miR-3570 expression compared with the transfection of control inhibitors (NC-i) (**Figure 2B** and **Supplementary Figure 1B**). Next, we tried to explore the effects of miR-210 mimics or inhibitors on the expression levels of inflammatory cytokines were investigated in LPS-stimulated MIC cells. NC, miR-210 and miR-3570 mimics, or NC-i, miR-210 and miR-3570 inhibitors were transfected into MIC cells for 48 h and treated with LPS for 6h before collecting cells for RNA extraction. The results showed that transfection of miR-210 and miR-3570 mimics could reduce expression levels of LPS-induced inflammatory cytokines such as TNF-α, IL-1β, and IL-8 (**Figure 2C**). In contrast, the miR-210 and miR-3570 inhibitors significantly upregulated the expression of inflammatory cytokines compared with control inhibitors (NC-i) (**Figure 2D**). In short, these data strongly confirmed that miR-210 and miR-3570 could negatively regulate the expression of inflammatory cytokines in MIC cells stimulated by LPS.

### miR-210 and miR-3570 Directly Target RIPK2

**Figure 3:**
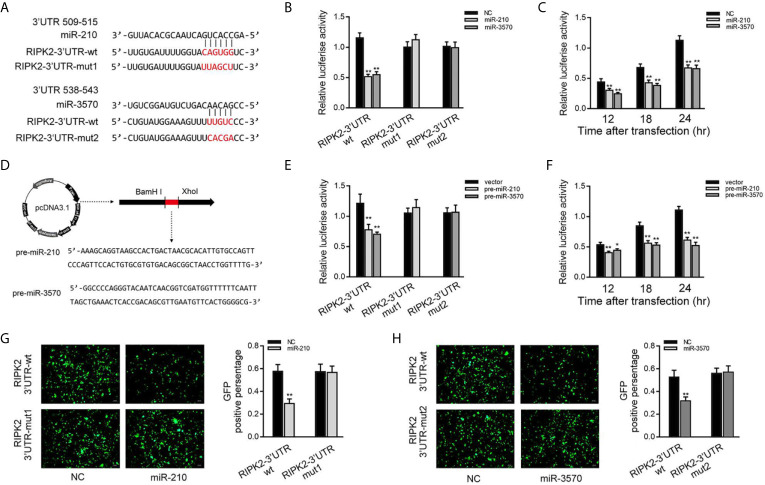
miR-210 and miR-3570 target the miiuy croaker RIPK2 gene. **(A)** Schematic diagram of miR-210 and miR-3570 and their predicted binding sites in RIPK2 3′UTR. **(B)** The dual luciferase reporter assay was applied to detect the regulation effect of miR-210 and miR-3570 mimics on wild-type RIPK2 3′UTR (RIPK2 3′UTR wt) or mutant-type of RIPK2 3′UTR (RIPK2-3′ UTR mut1/mut2). EPC cells were transfected with RIPK2-3′UTR wt (100 ng) or RIPK2-3′ UTR mut1/mut2 (100 ng), together with miR-210 (100 nM), miR-3570 (100 nM), or NC (100 nM) for 24 h, and then the luciferase activity was determined. **(C)** In EPC cells, the effect of miR-210 and miR-3570 on the luciferase activity of RIPK2 3′UTR was detected at different time points as indicated. **(D)** The pre-miR-210 and pre-miR-3570 sequences and schematic diagram for their plasmid. **(E)** EPC cells were contransfected RIPK2-3′UTR wt (100 ng) or RIPK2-3′UTR mut1/mut2 (100 ng), together with the pre-miR-210 plasmid (200 ng), pre-miR-3570 plasmid (200 ng) or pcDNA3.1 vector (200 ng). **(F)** The effect of pre-miR-210 plasmid, pre-miR-3570 plasmid on the luciferase activity of RIPK2-3′UTR was detected at different time points. Among them, pre-miR-210 and pre-miR-3570 plasmids were co-transfected into EPC cells with pcDNA3.1 vector. The luciferase activity value was achieved against the renilla luciferase activity. **(G, H)** EPC cells were co-transfected with the wild-type mVenus-RIPK2-3′UTR or mutant mVenus-RIPK2-3′UTR (100 ng), together with NC (100 nM), miR-210 (100 nM) **(G)**, and miR-3570 (100 nM) **(H)**. At 48 h posttransfection, the fluorescence intensity was evaluated by enzyme-labeled instrument (Thermo Scientific Varioskan LUX). the fluorescence intensity was observed by Leica DMiL8 fluorescence microscope. Scale bar, 20 μm; original magnification ×10. All luciferase activity was normalized to renilla luciferase activity. All data are presented as the means ± SE from three independent triplicate experiments. **p < 0.01; *p < 0.05 versus the controls.

Then, we verified whether RIPK2 is indeed the target of miR-210 and miR-3570. Through predictive analysis, it was found that the 509 to 543 bp nucleotides of RIPK2-3′UTR contained seed sequences that bind to miR-210 and miR-3570 (**Figure 3A**). To verify the reliability of the prediction results, the RIPK2-3′UTR sequence containing the original target sequence or mutant target sites were fused into the dual luciferase reporter vector to construct a dual luciferase reporter plasmid. Because the miRNA has the characteristics of highly conserved in different species (19). when miR-210 or miR-3570 mimics and luciferase reporter plasmid are cotransfected into EPC cells, miR-210 and miR-3570 mimics markedly reduced the luciferase activity of cells containing the wild-type RIPK2-3′UTR. On the contrary, the luciferase activity of cells transfected with mutant RIPK2-3′UTR was no affected (**Figure 3B**). Different time points experiment further showed that both miR-210 and miR-3570 could inhibit luciferase activity within 12 to 24 h after transfection, and the inhibition effect is the most significant at 24 h after transfection (**Figure 3C**). We then constructed the pre-miR-210 and pre-miR-3570 plasmids and transfected it into EPC cells (**Figure 3D**). As shown in **Figure 3E**, compared with the control group, pre-miR-210 and pre-miR-3570 decrease the luciferase activity of RIPK2-3′UTR without changing the luciferase activity of mutant RIPK2-3′UTR. The detection of pre-miR-210 and pre-miR-3570 showed the same effect as two miRNAs on the activity of RIPK2-3′UTR luciferase activity at different time points (**Figure 3F**). For further verification, wild-type or mutant type RIPK2-3′UTR was cloned into mVenus-C1 vector, and then co-transfected into EPC cells with miR-210 and miR-3570 mimics for 48 h. As shown in **Figures 3G, H**, the fluorescence intensity was significantly reduced in cells transfected with wild-type mVenus-RIPK2-3′UTR, indicating that both miR-210 and miR-3570 could down-regulate the expression of GFP gene. however, there was no change in fluorescence intensity in cells transfected with mutant-type mVenus-RIPK2-3′UTR. In conclusion, these data suggest that miR-210 and miR-3570 specifically target RIPK2 genes.

### miR-210 and miR-3570 Regulate RIPK2 Expression at the Posttranscriptional Level

**Figure 4:**
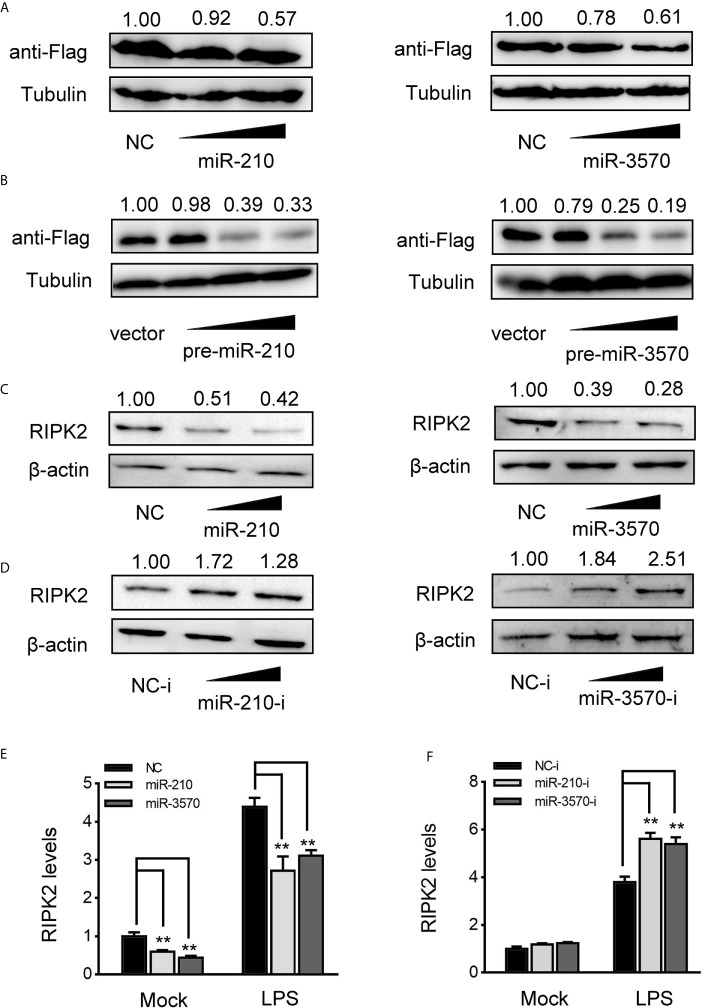
miR-210 and miR-3570 decrease the expression of RIPK2. **(A)** EPC cells were cotransfected with RIPK2 expression plasmid along with miR-210 mimics (0, 50, 100 nM), and miR-3570 mimics (0, 50,100 nM) or NC (100,50, 0nM) in EPC cells. After 48 h, RIPK2 protein levels were detected by western blotting and normalized to β-actin, respectively. **(B)** The concentrate gradient experiments were conducted for pre-miR-210 and pre-miR-3570 transfection. For the concentrate gradient experiments, pre-miR-210 plasmid (0, 50, 100, 200 ng) (left), pre-miR-3570 plasmid (0, 50, 100, 200 ng) (right), or pcDNA3.1 vector (200, 150, 100, 0 ng) along with RIPK2 expression plasmid cotransfected into EPC cells, After 48 h, expression of RIPK2 protein were assessed by western blotting, using β-actin as a loading control. **(C, D)** MIC cells were transfected with NC, miR-210 and miR-3570 **(C)**, or NC-i, miR-210-i and miR-3570-i **(D)**, after 48 h, RIPK2 protein levels were determined by western blotting and normalized to β-actin. **(E, F)** MIC cells contransfected with NC, miR-210 and miR-3570 **(E)** or NC-i, miR-210-i, and miR-3570-i **(F)** for 48 h, the MIC cells were treated with LPS for 6 h and RIPK2 mRNA expression was determined by qPCR and normalized to β-actin. The results are standardized to 1 in control cells. All data are presented as the means ± SE from three independent triplicate experiments. **p < 0.01 versus the controls.

MiRNAs function primarily degrade or inhibit mRNA translation by binding to the 3′UTR of target gene to achieve the goal of regulating gene expression post-transcriptional level. Therefore, to investigated whether miR-210 and miR-3570 are involved in the regulate of RIPK2 expression, we constructed RIPK2 expression plasmid with flag tag, and cotransfected with miR-210 and miR-3570 in EPC cells, respectively. As shown in **Figure 4A**, RIPK2 protein levels were decreased in a dose-dependent manner when overexpression of miR-210 and miR-3570. Similarly, pcDNA3.1 vector (vector), pre-miR-210, pre-miR-3570 and RIPK2 overexpression plasmid were co-transfected into EPC cells. compared with vector, pre-miR-210 and pre-miR-3570 plasmids significantly reduced RIPK2 protein levels (**Figure 4B**). Furthermore, we evaluated the regulatory effects overexpression of miR-210 and miR-3570 on endogenous RIPK2 in MIC cells. Compared with the NC, overexpressed miR-210 and miR-3570 down-regulated RIPK2 expression at the protein level (**Figure 4C**), whereas knockdown miR-210 and miR-3570 upregulated the expression of this protein (**Figure 4D**). To assess whether miR-210 and miR-3570 can also regulate the expression of endogenous RIPK2 at mRNA levels, MIC cells were transfected with miR-210, miR-3570 mimics and NC or miR-210, miR-3570 inhibitors and NC-i for 48 h and then stimulated the cells with LPS. The results indicate that miR-210 and miR-3570 mimics can reduce mRNA expression of RIPK2 (**Figure 4E**), on the contrary, miR-210 and miR-3570 inhibitors up-regulated the RIPK2 mRNA expression which significantly increased its expression following LPS stimulation 6 h (**Figure 4F**). Above all, these results suggested that miR-210 and miR-3570 negatively regulated the RIPK2 expression at both the protein and mRNA levels.

### miR-210 and miR-3570 Inhibit RIPK2-Induced NF-κB Signaling Pathway

**Figure 5:**
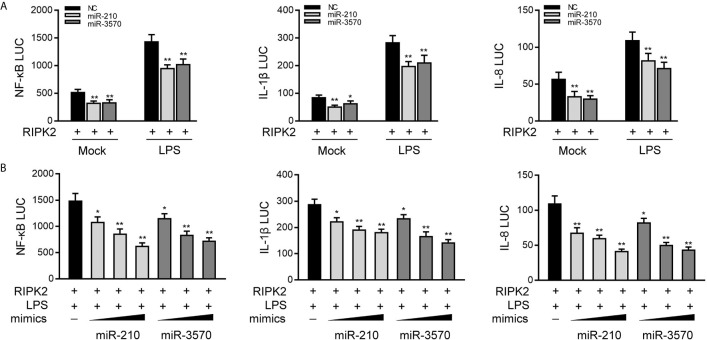
miR-210 and miR-3570 inhibit RIPK2-induced NF-κB signaling. **(A)** NC, miR-210, miR-3570 together with the pRL-TK renilla luciferase plasmid, NF-κB, IL-1β or IL-8 luciferase reporter gene and the RIPK2 expression plasmid were cotransfected with EPC cells for 24 h. Then the cells were stimulated with LPS for another 6 h and the luciferase activity was assessed. **(B)** miR-210 (0, 50, 100 nM) or miR-3570 (0, 50, 100 nM) in a concentration-gradient manner, together with luciferase reporter gene NF-κB, IL-1β and IL-8 were transfected with EPC cells for 24 h, the EPC cells were treated with LPS for 6 h, after which the luciferase activity value was determined by using the Dual-Luciferase reporter assay system. Luciferase activity was normalized to renilla luciferase activity. All data are presented as the means ± SE from three independent triplicate experiments. **p < 0.01; *p < 0.05 versus the controls.

Given that miR-210 and miR-3570 modulate the expression of inflammatory cytokines and negatively regulates RIPK2 expression, we thus examined whether miR-210 and miR-3570 affect RIPK2-mediated NF-κB signaling. We transfected EPC cells with the RIPK2 expression plasmid, together with the miR-210 and miR-3570 mimics or NC, and then stimulated the cells with LPS. The results revealed that both miR-210 and the miR-3570 mimics participated in the downregulation of the NF-κB, IL-1β, and IL-8 reporter genes (**Figure 5A**). The regulation of inhibition by miR-210 and miR-3570 was shown to be more significant in cells treated with LPS. Additionally, concentration gradient experiments of the miR-210 and miR-3570 were carried out. As shown in **Figure 5B**, miR-210 and miR-3570 showed a dose-dependent effect on the inhibition of the NF-κB, IL-1β, and IL-8 reporter genes. Collectively, the data as a whole suggest that miR-210 and miR-3570 participate in negatively regulating NF-κB signaling upon LPS stimulation in a manner dependent on modulating RIPK2.

### Knockdown of RIPK2 Inhibits Inflammatory Responses

**Figure 6:**
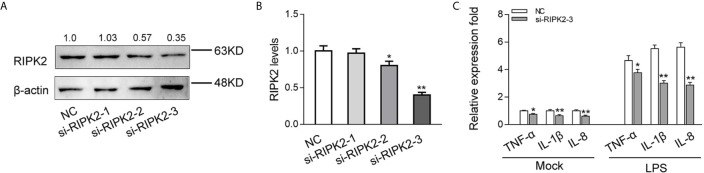
Knockdown of RIPK2 inhibits inflammatory responses. **(A, B)** Relative mRNA and protein levels of RIPK2 in MIC cells. si-RIPK2-1, si-RIPK2-2, si-RIPK2-3 or siRNA-control mimics (NC) by western blotting **(A)** and qPCR assays **(B)**, respectively. **(C)** At 48 h after transfected with NC or si-RIPK2-3, MIC cells were stimulated with LPS for 6 h. The mRNA expression levels for TNF-α, IL-1β, and IL-8 were determined and normalized to β-actin. All data are presented as the means ± SE from three independent triplicate experiments. **p < 0.01; *p < 0.05 versus the controls.

To explored the contribution of miiuy croaker RIPK2 in the innate immune response, we designed three RIPK2-specific small interfering RNAs (si-RIPK2-1/2/3) and transfected them in MIC cells. As shown in **Figures 6A, B**, the three siRNAs showed different degrees of inhibitory effects on mRNA and protein expression levels of RIPK2, Among, si-RIPK2-3 showed the most significant inhibitory effects than the si-RIPK2-1/2. Next, we silenced RIPK2 and examined the expression of inflammatory cytokine in MIC cells treated with LPS. As shown in **Figure 6C**, the mRNA expression levels of TNF-α, IL-1β and IL-8 were reduced by knockdown of RIPK2, which produced an effect similar to overexpression of miR-210 and miR-3570. These results further proved that miR-210 and miR-3570 can participate in the regulation of RIPK2-dependent signaling pathways and the production of inflammatory cytokines by suppressing endogenous RIPK2.

### miR-210 and miR-3570 Regulating RIPK2 Gene Is Found in Other Teleost

**Figure 7:**
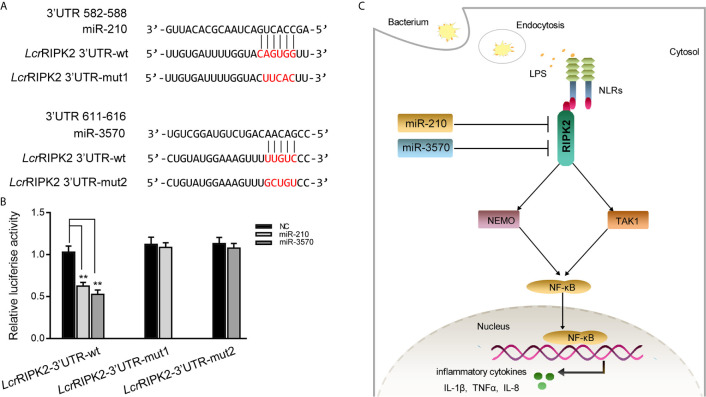
Proposed model for the mechanism regulated by miR-210 and miR-3570 in fish species. **(A)** Schematic alignment of the predicted target sites of miR-210 and miR-3570 in 3′UTR of *L. crocea* RIPK2 (*Lcr*RIPK2-3′UTR wt). **(B)** EPC cells were transfected with miR-210, miR-3570 or NC along with the wild-type or mutant *Lcr*RIPK2-3′UTR for 24 h and luciferase activity was determined. **(C)** The proposed model for the mechanism by which induced miR-210 and miR-3570 negatively regulate inflammatory cytokines production by targeting RIPK2 and inhibiting NF-κB signaling, thereby inhibiting excessive inflammatory response. All data are presented as the means ± SE from three independent triplicate experiments. **p < 0.01 versus the controls.

To illustrate the universality of the findings, we explored mechanisms of miR-210 and miR-3570 target RIPK2 in other fish species. *Larimichthys croaker* wild type RIPK2-3′UTR (*Lcr*RIPK2-3′UTR-wt) and containing target sites mutant of miR-210 and miR-3570 of RIPK2-3′UTR fragments (*Lcr*RIPK2-3′UTR-mut1/2) were cloned into pmir-GLO vector to construct the luciferase reporter gene (**Figure 7A**). The results showed that luciferase activity was decreased when miR-210 and miR-3570 mimics were co-transfected with *Lcr*RIPK2-3′UTR-wt plasmid into EPC cells, on the contrary, there was no effect on luciferase activity of *Lcr*RIPK2-3′UTR-wt transfected cells (**Figure 7B**).

## Discussion

Bacterial infection induces innate antibacterial immunity in host, and producing a series of interfering factors, such as cytokines and chemokines to cope with the invasion of pathogens. *V. harveyi*, belongs to the class of Gram-negative bacteria, and as an opportunistic pathogen that can infect fish and shellfish and is highly lethal to aquatic animals (32). Therefore, understanding the regulatory mechanisms of bacterial infection response is urgently needed for fish immunology research. However, due to the limitations of research techniques and methods, the research of fish immunology started late. In the previous reports, there are two components of Gram-negative bacteria, iE-DAP and LPS, which can be used as the specific ligands of fish cells immune response (7, 14). Among, compared with mammalian sensitivity to low concentration of LPS, fish cells are more tolerant to high concentration of LPS (39, 40). On the one hand, most teleost lack TLR4, which recognizes LPS, or the lack of auxiliary molecules MD2 and CD14 that cooperate with TLR4 to recognize LPS, resulting in teleost having higher tolerance to lipopolysaccharide (41, 42). On the other hand, the only known LPS-recognizing receptor in mammals has NRLs in addition to TLRs (43), whereas teleost fish lack TLR-like receptors relative to mammals and only have NOD-like receptors (44). Therefore, it is important to investigate the regulatory mechanisms of key molecules involved in NLR mediated signaling pathways in fish after bacterial infection.

NLRs, an important family of pattern recognition receptors, which exist in the cytoplasm and are mainly responsible for recognizing intracellular bacteria and invading viruses, then triggering immune responses (45). After identifying PAMPs, NLRs bind to the adapter protein RIPK2, and then transduction signals activate NF-κB and MAPK to regulate host cell response to the pathogen (46). However, overactivation of NLRs signal pathway can disrupt immune homeostasis and induce autoimmune diseases, which requires strict regulation by a series of endogenous mechanisms. Considering that NOD1/2 can transmit signals through RIPK2, and directly regulate the downstream adaptor protein RIPK2 of NLRs, it may be an effective way to avoid the occurrence of inflammatory diseases. Recently, a series of endogenous regulators that regulate RIPK2-mediated signaling pathways have been identified in mammals. Such as TNFR-related factors (TRAF2, TRAF5 or TRAF6), CYLD, BID, etc., through interaction with RIPK2 to negatively regulate specific transduction of NF-κB signaling events (47–49). Similarly, in teleost, zebrafish NLRC3-like1, by targeting RIPK2, inhibits the NOD1-RIPK2 pathway and thus NOD1 mediated antibacterial activity (12). ASC can interact with RIPK2 in Japanese medaka, inducing the downregulation of NF-κB activity (50). Therefore, endogenous regulatory molecules play an important role in mitigating the abnormal activation of signaling pathways caused by RIPK2 overactivation.

In recent years, an increasing number of studies has highlighted the function of microRNAs in the regulation of innate immune pathways. To study the function and mechanism of miRNAs in immune response, lentiviral infection, retroviral infection, plasmid transfection, transgene expression and miRNA mimics transfection approaches were used to study the function of miRNAs (51–53). Among them, miRNA mimics transfection is a common method in miRNA function research. In our study, we found that after transfection of miR-210 and miR-3570 mimics into MIC cells for 48 h, the abundance of miR-210 and miR-3570 increased by 40 to 50 times. Similarly, Jin et al., found that transfection of miRNA mimics into HeLa cells could increase the miRNAs content of cells hundreds of times (53). Interestingly, they found that this process accumulated a large number of high molecular weight RNA species, which could nonspecifically reduce the mRNA and protein levels of target genes in a short period of time (53). However, the nonspecific effects caused by the accumulation of high molecular weight RNA species diminish or disappear with extension of transfection time (53). Therefore, in our study, to avoid nonspecific effects of excessive accumulation of this high molecular weight RNA species on target gene expression, we prolonged the transfection time with miRNA mimics and to study the effect of miRNA on target genes. In teleost fish, many researches have shown that endogenous regulatory molecules miRNAs can regulate the immune response by regulating crucial immune-related genes, such as MITA, IRAK4, TBK1 (54–56). However, there are few reports on the regulation of RIPK2 by miRNAs. In this study, the target sequences of miRNAs were predicted to be exist in RIPK2 according to Targetscan (35), miRanda (36), and MicroInspector (37). Dual luciferase analysis showed that RIPK2 can serve as the target of miR-210 and miR-3570. Western blot showed that miR-210 and miR-3570 negatively regulate the expression of RIPK2 at the post-transcriptional level. Considering that the decrease of RIPK2 mRNA and protein levels cannot be completely excluded is due to non-specificity of the high molecular weight RNA species. Therefore, we further evaluated the biological functions of miRNAs by transfecting pre-miRNA. The results show that pre-miR-210 and pre-miR-3570 showed the same effect as miR-210 and miR-3570, and both could down-regulate RIPK2 expression at the post-transcriptional level. This is because pre-miRNA can mimic the biogenesis process of endogenous miRNAs, producing mature miRNAs without generating high molecular RNA species, thereby eliminating the effects of nonspecific effects (18). These results further indicate that miR-210 and miR-3570 can act as negative regulators to inhibit the expression of RIPK2 at the post-transcriptional level.

NF-κB is a crucial intracellular nuclear transcription factor, which plays a central role in the transcriptional regulation of cell information (57). It can participate in immune response and inflammatory response by regulating the expression of a variety of genes, such as IL-1β, TNFα (58, 59). In order to maintain immune homeostasis, multiple intracellular signal transduction adapters cooperate closely to regulate the NF-κB pathway, so as to avoid excessive inflammation and autoimmune diseases caused by long-term overactivation of NF-κB. Current studies have levels cannot reported that single miRNA regulates immune genes of TLRs and RLRs pathways, thereby regulating NF-κB signaling pathway activation in teleost fish. For example, miR-3570 regulates the activation of NF-κB in the TLRs and RLRs pathway by targeting miiuy croaker’s MyD88 and MAVS, respectively (33, 60). However, the regulatory effects of miR-210 and miR-3570 on the activation of NF-κB in the NLRs pathway has not been reported yet. Here, the dual luciferase reporting assay proved that miR-210 and miR-3570 could inhibit the NF-κB signaling pathway mediated by RIPK2. More importantly, we found that in LPS-stimulated MIC cells, miR-3570 and miR-210 inhibited the activation of NF-κB signal in the NLRs pathway by synergistically down-regulating RIPK2, thereby reducing the production of IL-1β, TNF-α and other inflammatory factors. Thus, we speculated that two miRNAs, miR-210 and miR-3570, could participate in the host antimicrobial immune response by downregulating NF-κB pathway activity.

In this study, we report that miR-210 and miR-3570 play a negative regulatory role in mediating LPS induced production of inflammatory cytokines TNF-α, IL-1β, IL-8. We found that miR-210 and miR-3570 in miiuy croakers could be rapidly upregulated upon *V. harveyi* and LPS treatment. Further studies revealed that RIPK2 could serve as a novel target of miR-210 and miR-3570, and the upregulated miR-210 and miR-3570 downregulated NF-κB signaling in teleost fish by targeting RIPK2 and thereby regulated the production of inflammatory factors (**Figure 7C**). This endogenous negative regulatory mechanism is essential for reducing pathogen infection induced inflammatory responses, not only enriching the regulatory network between microRNAs and mRNAs in teleost fish, but also providing new insights into miRNAs mediated regulatory mechanisms against pathogens.

## Data Availability Statement

The original contributions presented in the study are included in the article/**Supplementary Material**. Further inquiries can be directed to the corresponding authors.

## Ethics Statement

The animal study was reviewed and approved by Research Ethics Committee of Shanghai Ocean University.

## Author Contributions

Conceived and designed the experiments: TX. Performed the experiments: HS, RC, and WZ. Analyzed the data: HS and WZ. Contributed reagents/materials/analysis tools: HS, RC, WZ, and TX. Wrote the paper: HS and TX. All authors contributed to the article and approved the submitted version.

## Funding

This study was supported by the Open Fund of CAS Key Laboratory of Experimental Marine Biology, Institute of Oceanology, Chinese Academy of Sciences (KF2019NO1), National Natural Science Foundation of China (31802325, 31822057), and the National Key Research and Development Program of China (2018YFD0900503).

## Conflict of Interest

The authors declare that the research was conducted in the absence of any commercial or financial relationships that could be construed as a potential conflict of interest.

## Supplementary Material

Supplementary Figure 1 miR-3570 negatively regulate expression of inflammatory cytokine. **(A, B)** the miiuy croaker MIC cells were transfected with NC or miR-3570 mimics (miR-3570) **(A)** and miR-3570 inhibitors (miR-3570-i) and control inhibitors (NC-i) **(B)**. **(C, D)** After transfection of miR-3570 or NC **(C)** and miR-3570-i or NC-i **(D)** for 48 h. The MIC cells were then stimulated with LPS for 6h, the mRNA expression levels of TNF-α, IL-1β, and IL-8 were analyzed by qPCR. Those data were normalized to β-actin. Results are standardized to 1 in control cells. All data are presented as the means ± S.E. (error bars) from three independent triplicate experiments. **p < 0.01; *p < 0.05 versus the controls.
