# Supplementary figures and images for: microRNA-210 and microRNA-3570 Negatively Regulate NF-κB-Mediated Inflammatory Responses by Targeting RIPK2 in Teleost Fish
Source: Front Immunol. 2021 Mar 31;12:617753. doi: 10.3389/fimmu.2021.617753 (PMC8044448; doi:10.3389/fimmu.2021.617753)

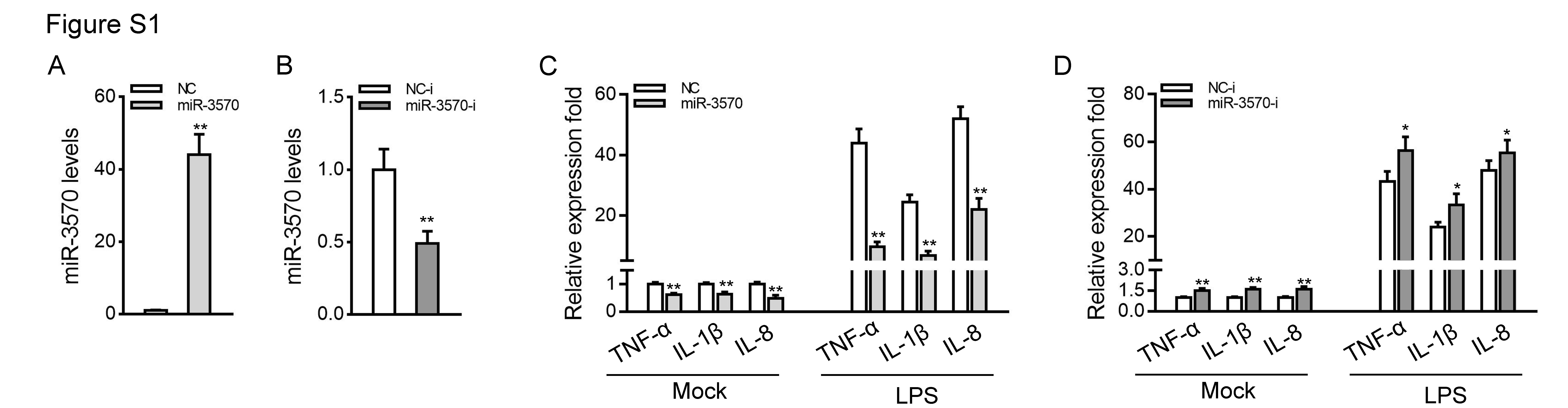

Supplement: Supplementary Figure 1 — miR-3570 negatively regulate expression of inflammatory cytokine. (A, B) the miiuy croaker MIC cells were transfected with NC or miR-3570 mimics (miR-3570) (A) and miR-3570 inhibitors (miR-3570-i) and control inhibitors (NC-i) (B). (C, D) After transfection of miR-3570 or NC (C) and miR-3570-i or NC-i (D) for 48 h. The MIC cells were then stimulated with LPS for 6h, the mRNA expression levels of TNF-α, IL-1β, and IL-8 were analyzed by qPCR. Those data were normalized to β-actin. Results are standardized to 1 in control cells. All data are presented as the means ± S.E. (error bars) from three independent triplicate experiments. **p < 0.01; *p < 0.05 versus the controls. [file Image_1.jpeg]
