# Supplementary material for: microRNA-210 and microRNA-3570 Negatively Regulate NF-κB-Mediated Inflammatory Responses by Targeting RIPK2 in Teleost Fish
Source: Front Immunol. 2021 Mar 31;12:617753. doi: 10.3389/fimmu.2021.617753 (PMC8044448; doi:10.3389/fimmu.2021.617753)
Supplement: Supplementary file 2 [file Table_1.docx]

**Supplementary Table S1** PCR primer sequence information in this study.

| **Primers** | **Sequences (5’-3’)** | **Application** |
| --- | --- | --- |
| **Vector construction** | | |
| RIPK2-3’UTR-1F | CGCGAGCTCCGCACCGCCACTGTCTATGT | Amplification of RIPK2-3’UTR |
| RIPK2-3’UTR-1R | TGCTCTAGACGTACTTCTCATTCTGGGTG |  |
| RIPK2-3’UTR-GFP-1F | CCGAAGCTTGCTAGCATTTGGCTTTGAGGTTA | Amplification of RIPK2-3’UTR |
| RIPK2-3’UTR-GFP-1R | CGCGGATCCCTTCTCATTCTGGGTGTT |  |
| RIPK2-3’UTR-MT1-1F | TGGTATTAGCTTCATGTGACTGTATGGAAAGTTTTTG | Mutation of RIPK2-3’UTR |
| RIPPK2-3’UTR-MT1-1R | CACATGAAGCTAATACCAAAATCACAACTCATTCCAAC |  |
| RIPK2-3’UTR-MT2-1F | AAGTTTCACGACCTGCCTGGGCTCTTTTTTTC | Mutation of RIPK2-3’UTR |
| RIPK2-3’UTR-MT2-1R | GGCAGGTCGTGAAACTTTCCATACAGTCACATGACCA |  |
| *Lcr*RIPK2-3’UTR-1F | CTAGCTAGCCCAAATGACGGAAACAAT | Amplification of *L. crocea* RIPK2-3’UTR |
| *Lcr*RIPK2-3’UTR-1R | CCGCTCGAGGAATGAACATCAAACCCT |  |
| *Lcr*RIPK2-3’UTR-MT1-1F | GTCAGGCTTCACTTTGAGTAGATGTGTTGCATG | Mutation of *L. crocea* RIPK2-3’UTR |
| *Lcr*RIPK2-3’UTR-MT1-1R | CTCAAAGTGAAGCCTGACTAAATAAATCACCTCATTAAATCCATAACA |  |
| *Lcr*RIPK2-3’UTR-MT2-1F | AAGTTTGCTGTCCTGCCTGGGCTCTTTTTTTT | Mutation of *L. crocea* RIPK2-3’UTR |
| *Lcr*RIPK2-3’UTR-MT2-1R | GGCAGGACAGCAAACTTTCCATACAGTCACATAACCAC |  |
| pre-miR-3570-1F | CGCGGATCCGTGGTTTGGAAGCTGGAA | Amplification of pre-miR-3570 |
| pre-miR-3570-1R | CCGCTCGAGAAGCAGACTGTCATCCCT |  |
| pre-miR-210-1F | CCCAAGCTTGGTAAGCCACTGACTAACGC | Amplification of pre-miR-210 |
| pre-miR-210-1R | CCGGAA TTCACA TGAACACCACTGGGAGA |  |
| **qPCR** | | |
| miR-3570-RT-F | AGTACAATCAACGGTCGATG | Expression of miR-3570 |
| miR-3570-RT-R | GTCCAGTTTTTTTTTTTTTTTAAACCA |  |
| miR-210-RT-F | GCCACTGACTAACGCACA | Expression of miR-210 |
| miR-210-RT-R | GGTCCAGTTTTTTTTTTTTTTTCAA TG |  |
| 5.8S-RT-F | AACTCTTAGCGGTGGATCA | Expression of 5.8s rRNA |
| 5.8S-RT-R | GTTTTTTTTTTTTTTTGCCGAGTG |  |
| TNF-α-RT-F | GTTTGCTTGGTACTGGAATGG | Expression of TNFα |
| TNF-α-RT-R | TGTGGGATGATGATCTGGTTG |  |
| IL-1β-RT-F | CATAAGGATGGGGACAACGAG | Expression of IL-1β |
| IL-1β-RT-R | TAGGGGACGGACACAAGGGTA |  |
| IL-8-RT-F | AGCAGCAGAGTCTTCGT | Expression of IL-8 |
| IL-8-RT-R | TCTTCGCAGTGGGAGTT |  |
| RIPK2-RT-1F | GCTGTCCAGGCAGATTCCAT | Expression of RIPK2 |
| RIPK2-RT-1R | GGCGTTCGTCAGGGTTAGAG |  |
| β-actin-RT-F | GAGCCGCACGCTTCTTT | Expression of GAPDH |
| β-actin-RT-R | CTGCTGTAGCCGAGGAC |  |
